# Decreasing child death from diarrhoea requires more focus on poor hygiene

**DOI:** 10.7189/jogh.10.020386

**Published:** 2020-12

**Authors:** Hugo M Smeets, Fatoumata S Keìta

**Affiliations:** 1 Julius Center for Health Sciences and Primary Care, University Medical Center Utrecht, Utrecht, the Netherlands; 2 Centre de Santé, Fougou, Prefecture Mali, Guinée

Children in low-income countries under the age of 5 years have a high mortality risk. If, with good luck, they survived the neonatal life-threatening diseases during the first month, they have a chance of 16% to die from diarrhoea in the next 59 months [1]. The recently in the Lancet Infection Disease published studies of the Global Burden of Disease project demonstrated, that in 2015 in low-middle income countries still more than half a million children died from diarrhoea [2]. Although this was a decrease of 57% from the year 2000, the United Nations Millennium Development Goal of a reduction towards 66% was not yet achieved. If that had been the case, the chance of dying would be reduced to 10%. With the current trend of 6.5%, there is hope this goal will soon be achieved. Unfortunately, this progressive decline will be inconsistent for Sub-Saharan Africa. Due to the large population growth, in 2030 the absolute number of children who die from diarrhoea will be higher than today and a large proportion (72%) of these children will die in the first two years of life [3,4]. In contrast to the decline of deaths, it is remarkable that the number of diarrhoea disease-episodes between 1990 and 2010 barely decreased. This dropped from 1,9 to 1,7 billon, a modest number of 10.5% [5,6]. Why is there such a big difference in decrease between mortality and incidence ?

In the international literature the discussion to decrease mortality emphasizes often on the implementation and the improvement of accessible public health care. Vaccination programs for rotavirus for examples could prevent 28% of diarrheal death [7]. Knowing that most children die before the second birthday and that the risk for infection remains high, we have to realise that because of poor infrastructures and costs only the severe cases become visible in health care. Looking more precisely at the causes of death due to diarrhoea two elements seem the most important. First, the risk of death usually depends on concomitant infections, of which pneumonia, malaria and measles are the most common. In children with a weak condition, this multi-causal effect is disastrous. Too little breastfeeding, poor nutrition, exhaustion of vitamin A and Zinc gives them too little resistance to survive two or more infections simultaneously. The second is the chain of factors that leads to diarrhoea. In addition to the poor condition, it is the poor general environmental conditions that cause unfavourable effects. A poor and cramped housing, missing sanitary facilities, no running water and little hygiene for the body and food preparation undeniably increase the chance of infection. All in all, the conclusion is that over the past decades the development of public health medical facilities has improved in successfully treating diarrhoea patients with concomitant infections. This development has significantly reduced mortality [8]. However, the chance of infection has remained high, despite all the efforts of programs that want to improve water and sanitation hygiene, for example. Evaluations describe that these programs are less effective and sustainable over a more extended period, with the result that the number of episodes could have decreased to a much lesser extent.

**Figure:**
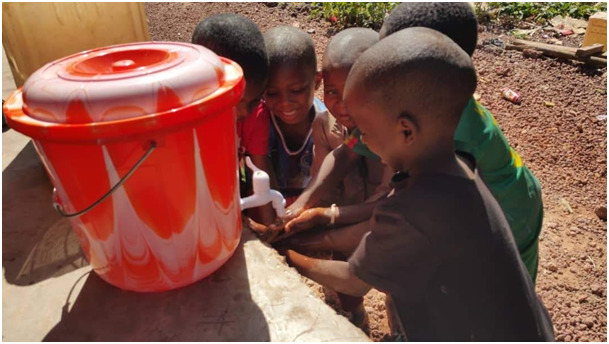
Photo: Children learning to wash hands before eating, as a practical part of a prevention program for hygiene promotion (from the authors’ own collection, used with permission).

To expand better health care is a logical and effective reflex. A more rational approach is to break the vicious circle by promoting prevention and to give first attention to improvement of basic hygienic behaviour. To diminish the number of deaths is important, but to prevent sickness as well. Often and long-lasting diarrhoea causes growth impairment and stunting which impedes physical and mentally child development in general [9]. To break the constant burden on health due to diarrhea, a lot of prevention is being done, such as the Wash and Shine programs. Nevertheless, these were insufficiently effective in overall analyses [10]. Partly because technical maintenance was often required, partly because the changes did not last in the long term. Regarding the area of prevention, rather simple and inexpensive ways of improving water and food hygiene must be inquired. It is highly necessary to develop methods that aim at fundamental behavioural change, and that can apply in the long term. Something we also deduced after a six-month observation in rural West Africa [10-12] What is most striking is the garbage all over in the streets and the lack of sanitation. A critical risk factor for diarrhoea is that all children play without supervision in the dirt of waste and faeces of animals in their compound. We never noticed those washing hands with soap before eating. Indeed, people wash themselves in the morning after a toilet visit, starting with their feet and directly, without soap, to continue with their face, ears and teeth. In fact dirt and dust are not really removed. Another example is that the butcher slaughters his animals outside on a wooden table that black sees of clotted blood and flies. And dust is everywhere in the street, houses, shops, restaurants. To substantiate subjective conclusions from these for diarrhoea highly infectious situations, we carried out a structured interview. With the help of a small health centre in Fougou, a community of 22 427 inhabitants in North Guinea we randomly selected 19 women, 29 years on average, who visited the centre in the last six months with their child (<5 years) suffering from diarrhoea. Visiting their homes we notices that two-thirds of them was illiterate and 60% had no water resource on their compound. However they said to use soap washing their child daily. Half of the women did not recognise the symptoms of diarrhoea and how to nurse their sick child. With the causes of diarrhoea 85% was unknown and 65% had no idea of prevention measures. After consulting the doctor 40% had the intention to practice better hygiene with their child. Conclusively the knowledge of women in rural Guinea about diarrhoea and how to prevent and control it is minimal. 

After the Ebola outbreak, a prevention program was rolled out in Guinea to prevent spread. However, this information about “hand washing” was soon forgotten after the epidemic stopped. If adherence to short-term information intervention does not get caught up in a behavioural change, the question is which approach can pay off more efficacy [13]. Concerning primary prevention, more is necessary for a behavioural change than an awareness-raising conversation. A concrete interest of the mother, such as her sick child that threatens to die or will cost a lot of money for medication, could be a start. It is also effective to go from door to door, which is feasible together with ongoing vaccination programs. Three to four times a year, vaccinations take place, whereby every mother can again be given attention about prevention of diarrhoea and repeat information about hygiene is possible. Distribution of useful material is a requirement, such as factsheets about causes, prevention and adequate nutrition for diarrhoea. In every vaccination-rounds these sheets can be recalled in the memory and one can also ask directly about the experiences with the advices and how they were followed up. Sustainability, that hand washing with soap is ‘normal’, is only achievable when feelings of disgust about dirty hands have been internalized. Emphasizing the chance that one in six children will die, unless there is compliance with adequate hygiene, can trigger change.
